# Daily Costs and Cost Effectiveness of Glaucoma Fixed Combinations in China

**DOI:** 10.1155/2020/2406783

**Published:** 2020-07-30

**Authors:** Chenjia Xu, Ruru Guo, Dandan Huang, Jian Ji, Wei Liu

**Affiliations:** ^1^Tianjin Key Laboratory of Retinal Functions and Diseases, Tianjin International Joint Research and Development Centre of Ophthalmology and Vision Science, Eye Institute and School of Optometry, Tianjin Medical University Eye Hospital, Tianjin 300384, China; ^2^Department of Ophthalmology, University of Groningen, University Medical Center Groningen, Groningen, Netherlands

## Abstract

**Background:**

The aim of this study was to compare the daily costs and cost effectiveness of fixed combination glaucoma drugs in China.

**Methods:**

This study included the following fixed combination drugs: brinzolamide 1% and timolol 0.5% (Azarga; Alcon, Inc., Fort Worth, TX, USA), travoprost 0.004% and timolol 0.5% (DuoTrav; Alcon, Inc.), bimatoprost 0.03% and timolol 0.5% (Ganfort; Allergan, Inc., Dublin, Ireland), and latanoprost 0.005% and timolol 0.5% (Xalacom; Pfizer, Inc., New York, NY, USA). Five bottles of each drug were measured. The mean actual volume, mean actual number of drops, volume per drop, daily cost, yearly cost, and per mmHg reduction cost for each drug were calculated.

**Results:**

The volumes per drop ranged from 32.61 ± 2.90 *μ*l (DuoTrav) to 24.38 ± 0.23 *μ*l (Ganfort). The number of usage days per bottle varied from 36 days (DuoTrav) to 61 days (Ganfort). Azarga had the lowest daily cost ($0.23) and yearly cost ($84.72), while DuoTrav had the highest daily cost ($0.79) and yearly cost ($287.02). Azarga costed $2.17–$3.30 per mmHg intraocular pressure reduction, which was lower than the other three drugs. For the prostaglandin and *ß*-adrenergic blocker FCs, Ganfort had the lowest daily cost ($0.35) and per mmHg reduction cost (from $3.40 to $4.04).

**Conclusions:**

The daily costs of these drugs were significantly different, with Azarga having the lowest daily cost and best cost effectiveness. For the prostaglandin and *β*-adrenergic blocker fixed combinations, Ganfort was the most economical choice with its lower daily cost and per mmHg reduction cost. The results of this study could provide drug selection guidance from an economic perspective, but various factors should be considered when making a decision.

## 1. Introduction

Glaucoma is a disease characterized by the thinning of the optic nerve fiber layer, a defect in the corresponding visual field, and irreversible blindness. A high intraocular pressure (IOP) plays a major role in the onset and progression of glaucoma, and it is the main controllable factor for glaucoma [1, 2]. IOP-lowering eye drops, due to their convenience, effectiveness, and noninvasiveness, continue to be the initial therapy of choice for most glaucoma patients. However, the cost of medications has become not only the main risk factors for poor compliance in patients with glaucoma [3, 4] but also an important national issue. Variations in costs among antiglaucoma medications may affect decisions by managed care administrators, as well as prescribing clinicians, and can cause a high impact in public health budget [5–8]. When considering the fact that glaucoma patients often use medications for a long time, the cost estimations of different IOP-lowering eye drops are very important and meaningful, especially for a country with a large population density and a great number of glaucoma patients, such as China. Currently, there are several antiglaucoma medications available, including prostaglandins, *β*-adrenergic blockers, *α*-adrenergic agonists, cholinergics, carbonic anhydrase inhibitors, and the newly introduced fixed combinations (FCs). FCs can reduce the flushing effect between different drugs, decrease the total usage frequency of drugs, and minimize the damage to the ocular surface caused by glaucoma medications and preservatives [9], and thus improve compliance. At present, several researchers from different countries have compared the daily costs of various antiglaucoma medications [10–13], but FCs were not included in most of the published papers. In this study, we aimed to compare the daily costs and cost effectiveness of the FCs currently available in China.

In China, from the data of National Medical Products Administration (http://www.app1.sfda.gov.cn) at January 2, 2020, five FCs are available, including brinzolamide 1% and timolol 0.5% (Azarga; Alcon, Inc., Fort Worth, TX, USA), travoprost 0.004% and timolol 0.5% (DuoTrav; Alcon, Inc.), bimatoprost 0.03% and timolol 0.5% (Ganfort; Allergan, Inc., Dublin, Ireland), latanoprost 0.005% and timolol 0.5% (Xalacom; Pfizer, Inc., New York, NY, USA), and brimonidine 0.2% and timolol 0.5% (Combigan; Allergan, Inc., Dublin, Ireland). However, Combigan is available in only one or two hospitals in China and was not enrolled for analysis in our current study. The price of Ganfort and Xalacom is uniform in China, $21.55 and $27.23, respectively. The price of Azarga and DuoTrav varies in different provinces, and we collected all the prices of Azarga and DuoTrav, and the median value was used for analysis (Azarga, $10.34, ranged from $9.83 to $10.36; DuoTrav, $28.23, ranged from $27.52 to $32.32).

## 2. Materials and Methods

The Azarga, DuoTrav, Ganfort, and Xalacom FCs that were included in this research were ordered from wholesalers. The DuoTrav and Xalacom were labeled as containing 2.5 ml per bottle. Each bottle of Ganfort was labeled 3 ml, and each bottle of Azarga was labeled 5 ml. Five bottles of each medication (all imported oversea) were chosen randomly, and they were kept at a suitable temperature before the test.

For each test, the bottles were left to stand for a while at 25°C until the liquid in the bottle did not flow. After the bottle was rotated 180°, the same researcher slowly squeezed the bottle to ensure that only one drop dropped out; then, the bottle was put back for a while to stop the liquid from flowing. This process was repeated until the bottle was empty. The drops were collected in a 5 ml tube with increments of 0.1 ml, and each number of drops was calculated in the same environment. The researcher ensured that the tube was clean and dry before the next bottle measurement. Afterwards, the mean actual volume and the mean actual number of drops for each drug were obtained. The mean volume per bottle divided by the mean number of drops per bottle gave the mean volume per drop.

Assuming that these drugs were used in both eyes and that one drop of each drug could be successfully dropped into the conjunctival sac each time, the number of usage days per bottle was calculated by dividing the mean number of drops per bottle by the number of drops per day for both eyes. The price of each drop was calculated by dividing the unit price of each bottle by the mean number of drops per bottle. The price per drop multiplied by the number of drops per day equaled the price per day for each medication. The price per year for each drug was calculated by multiplying the price of the drug per day by 365 days per year.

Cost effectiveness was calculated by dividing the drug cost by the degree of the IOP reduction (mmHg), which is considered the efficacy of the drug, over the course of the study period [14]. The price per day of each drug multiplied by the number of days studied equals the drug cost during the study. The ranges of IOP reduction of glaucoma fixed combinations were obtained from published data [15–18]. In order to ensure the homogeneity of different studies, studies with similar study period (about 3 months) were selected. Furthermore, all the selected studies were prospective, randomized, double-masked, or evaluator-masked parallel group clinical trials. The participants were mostly open-angle glaucoma or ocular hypertension patients, and all the IOP were measured by Goldmann applanation tonometry.

Analysis of variance (ANOVA) was used to compare daily cost among the groups. The post hoc least significant difference (LSD) test was used for multiple comparisons. A *P* < 0.05 was considered statistically significant. The SPSS 17.0 software (SPSS Inc., Chicago, IL, USA) was used for all statistical analyses.

## 3. Results

The mean actual volume, mean actual number of drops, mean volume per drop, and number of usage days per bottle are shown in Table 1. The actual volume per bottle was a bit different from the labeled volume. The mean actual volume per bottle of DuoTrav and Xalacom was 2.33 ml and 2.66 ml, respectively, which was labeled as 2.5 ml. The mean actual volume per bottle of Ganfort and Azarga was 2.98 ml and 4.71 ml, respectively, while the labeled volume was 3.0 ml and 5.0 ml, respectively. The mean actual number of drops ranged from 71.8 ± 4.15 (DuoTrav) to 178.2 ± 3.19 (Azarga). The largest volume per drop was 32.61 ± 2.90 *μ*l (DuoTrav), and the smallest volume per drop was 24.38 ± 0.23 *μ*l (Ganfort).

The number of drops per day, usage time per bottle, price per bottle, daily cost, and yearly cost of each drug are shown in Table 2. The number of usage days per bottle ranged from 36 days (DuoTrav) to 61 days (Ganfort). The unit price of these drugs ranged from $10.34 (Azarga) to $28.23 (Xalacom). Azarga had the lowest daily cost ($0.23) and yearly cost ($84.72), while DuoTrav had the highest daily cost ($0.79) and yearly cost ($287.02). For prostaglandin and *β*-adrenergic blocker FCs, Ganfort had the lowest daily cost ($0.35) and yearly cost ($128.53). There is a statistically significant difference in daily cost among these medications (ANOVA, *P* < 0.01; LSD, *P* < 0.01).

The detailed study information from the published papers and the calculated cost effectiveness results are shown in Table 3. All the studies lasted about three months, and the baseline IOP was similar. Xalacom had the largest IOP reduction (ranged from 6.3 mmHg to 12.5 mmHg), and Azarga yielded a range of cost effectiveness from $2.17 to $3.30 per mmHg decrease in IOP, indicating Azarga was more cost effective than DuoTrav, Ganfort, and Xalacom. For the prostaglandin and *β*-adrenergic blocker FCs, Ganfort had the lowest per mmHg reduction cost (from $3.40 to $4.04).

## 4. Discussion

In this study, we aimed to compare the daily costs and cost effectiveness of different FCs in China, and we enrolled all of the most commonly used FCs available in China. Our results showed that the daily costs of these drugs were significantly different. Azarga had the lowest daily cost ($0.23), followed by Ganfort ($0.35) and Xalacom ($0.56). DuoTrav had the highest daily cost ($0.79), which was more than three times that of Azarga. For the prostaglandin and *β*-adrenergic blocker FCs, Ganfort had the lowest daily cost, which was consistent with previous studies [19]. This might be the result of the lower price ($21.55), the larger labeled volume (3.0 ml) and actual volume (2.98 ± 0.02 ml) per bottle, and the less volume (24.38 ± 0.23 *μ*l) per drop of Ganfort. For the yearly cost, the variation among the different medications was much larger. DuoTrav had the highest yearly cost ($287.02), which was twice as much as Ganfort ($128.53), and the difference in the yearly cost between Azarga and DuoTrav was $202.30.

In this study, we found that Ganfort had the least drop volume (24.38 ± 0.23 *μ*l), followed by Azarga (26.45 ± 0.41 *μ*l) and Xalacom (27.22 ± 0.87 *μ*l), while DuoTrav had the largest drop volume (32.61 ± 2.90 *μ*l). This may have been the result of different dropper tips and bottle designs and characteristics, as well as the different physicochemical properties of each medication, which will determine the drop size [20]. However, the drop sizes of the same medication were also different in different studies. Xalacom was reported to have 81.00 ± 0.97 drops per bottle, and the drop volume was 30.87 ± 0.37 *μ*l [13]. In another study, the drops per bottle and the drop volumes were reported to be 103 ± 2.49 and 0.030 ± 0.000 ml for Ganfort and 90 ± 7.58 and 0.030 ± 0.003 ml for Xalacom, respectively [19], which were both different from our study. The drop volumes of Ganfort (24.38 ± 0.23 *μ*l) and Xalacom (27.22 ± 0.87 *μ*l) in our study were both less than those of previous studies [13, 19]. This may be related to the squeezing procedure, which is another drop size-determining factor. In one study, 5 researchers squeezed the bottles, and the operational errors from these different researchers might have created variations in the number of drops of the same medication [11]. In another study, each bottle was held at approximately 45°, which might have increased the volume of the drug remaining in the mouth of the bottle [21]. Therefore, in this study, all of the bottles were squeezed by one researcher, and all of the bottles were rotated 180°, squeezed slowly, and put back to avoid waste, minimize the volume of each drop, and maximize the number of drops. However, in the real world, drug waste can be induced by many factors, such as bottle direct missing, bottle squeezing, blinking, poor dropper tip visibility, and shaky hands [22], especially in elderly patients. Therefore, with the increasing age of glaucoma patients, drug waste will increase accordingly [23, 24]. In order to reduce waste and decrease the daily costs of medications, patients should be instructed to use eye drops correctly, and the design of the bottle and tip should be improved by manufacturers in order to minimize the drop volume.

In order to evaluate the cost effectiveness of each drug, the information from published papers was used. Although they were from different trials, the study period and the IOP baseline was similar, and the reduction of IOP was also similar. Holló et al. had compared a variety of studies and confirmed that DuoTrav, Ganfort, and Xalacom had the similar effects in IOP reduction [25]. According to our results, the cost effectiveness of Azarga is the best. Ganfort costs 3.40–4.04 $/mmHg decrease in IOP and had the best cost effectiveness in the combination of prostaglandins and *β*-adrenergic blockers, which is consistent with a study from Spain [26].

Nowadays, glaucoma FCs are becoming more and more recognized and accepted by the public. Although FCs can effectively reduce the IOP, decrease the drug use frequency, and relieve ocular surface damage, the costs of FCs are higher [19]. The costs of medications have been proven to be a major concern in drug selection, and it is recommended to communicate with patients at the time of prescribing in order to improve medication compliance, especially in developing countries [27, 28].

We acknowledge the limitations of our study. First, this study was based on a best-case scenario, which did not involve the expiration dates of the drugs, daily wastes of the drugs, drug supplementation frequency, and ineffectiveness of the drugs. Second, the price of medications may change, although the standard market price in China for each medication is regulated by national agency. However, the results of our study, especially the average number of drops per bottle or average number of days for usage per bottle, may be useful to recalculate an updated daily cost when the price changes.

## 5. Conclusions

In conclusion, the daily costs of FCs were significantly different, with Azarga having the lowest daily cost and best cost effectiveness. For the prostaglandin and *β*-adrenergic blocker fixed combinations, Ganfort was the most economical choice with its lower daily cost and per mmHg reduction cost. To the best of our knowledge, our study is the first to compare the daily costs and cost effectiveness of all of the FCs available in China, which should provide guidance for drug selection in the future.

## Figures and Tables

**Table 1:** The mean actual volume, the mean actual number of drops, the mean volume per drop, and the number of usage days per bottle of glaucoma fixed combinations.

Drug	Labeled volume/bottle (ml)	Actual mean volume/bottle (ml)	No. of drops/bottle (95% CI)	Volume/drop (*μ*l) (95% CI)
Azarga	5.0	4.71 ± 0.06	178.2 ± 3.19 (174.23–182.17)	26.45 ± 0.41 (25.93–26.96)
DuoTrav	2.5	2.33 ± 0.08	71.8 ± 4.15 (66.65–76.95)	32.61 ± 2.90 (29.01–36.22)
Ganfort	3.0	2.98 ± 0.02	122.4 ± 0.55 (121.72–123.08)	24.38 ± 0.23 (24.09–24.67)
Xalacom	2.5	2.62 ± 0.03	96.4 ± 2.40 (93.05–99.75)	27.22 ± 0.87 (26.13–28.30)

95% CI: 95% confidential interval.

**Table 2:** The number of drops per day, the usage time per bottle, the price per bottle, the daily cost, and the yearly cost of glaucoma fixed combinations.

Drug	Dose**∗**/d (ou)	No. drops/d (ou)	No. d/bottle (ou)	Cost/bottle (US Dollar^**†**^)	Cost/drop (US Dollar^**†**^)	Cost/d (ou) (US Dollar^**†**^)	Cost/year (ou) (US Dollar^**†**^)
Azarga	BID	4	45	10.34	0.06	0.23	84.72
DuoTrav	QD	2	36	28.23	0.40	0.79	287.02
Ganfort	QD	2	61	21.55	0.18	0.35	128.53
Xalacom	QD	2	48	27.23	0.28	0.56	206.20

∗BID: twice per day; QD: once per day. ^†^1 US dollar = 6.9614 Yuan (based on the exchange rate at January 2, 2020).

**Table 3:** The cost effectiveness of glaucoma fixed combinations.

Drug	Study	Study period (days)	Sample size	Age (years)	Gender, female (%)	Baseline IOP (mmHg)	IOP reduction (mmHg)	Total cost (US dollar)	Cost effectiveness ($/mmHg decrease in IOP)
Azarga	Sezgin et al.	92	57	54.8 ± 8.5	56.1	24.6–29.9	6.42–9.74	21.16	2.17–3.30
DuoTrav	Schuman et al.	90	155	62.4 ± 10.9	59.4	22.9–25.6	7.0–8.2	71.10	8.67–10.16
Ganfort	Goldberg et al.	84	283	63.5 (23–86)	57.2	23.8–25.4	7.27–8.55	29.40	3.40–4.04
Xalacom	Shin et al.	92	125	64.0 ± 11.0	53.6	27.9 ± 3.6	9.4 ± 3.1	51.52	4.12–8.18

## Data Availability

The datasets used and/or analysed during the current study are available from the corresponding author upon reasonable request.
